# Adipose tissue lymphocytes and obesity

**DOI:** 10.20517/jca.2023.38

**Published:** 2023-12-31

**Authors:** Feng Gao, Benjamin Litchfield, Huaizhu Wu

**Affiliations:** Department of Medicine, Baylor College of Medicine, Houston, TX 77030, USA.

**Keywords:** Obesity, adipose tissue, insulin resistance, T cells, B cells

## Abstract

Obesity is associated with chronic inflammation in adipose tissue (AT), mainly evidenced by infiltration and phenotypic changes of various types of immune cells. Macrophages are the major innate immune cells and represent the predominant immune cell population within AT. Lymphocytes, including T cells and B cells, are adaptive immune cells and constitute another important immune cell population in AT. In obesity, CD8+ effector memory T cells, CD4+ Th1 cells, and B2 cells are increased in AT and promote AT inflammation, while regulatory T cells and Th2 cells, which usually function as immune regulatory or type 2 inflammatory cells, are reduced in AT. Immune cells may regulate the metabolism of adipocytes and other cells through various mechanisms, contributing to the development of metabolic diseases, including insulin resistance and type 2 diabetes. Efforts targeting immune cells and inflammation to prevent and treat obesity-linked metabolic disease have been explored, but have not yielded significant success in clinical studies. This review provides a concise overview of the changes in lymphocyte populations within AT and their potential role in AT inflammation and the regulation of metabolic functions in the context of obesity.

## Graphical Abstract

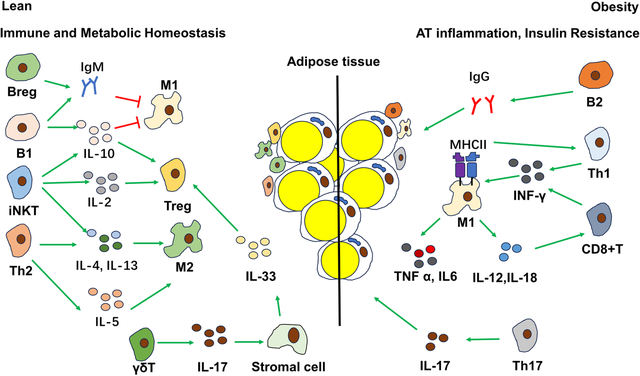

## INTRODUCTION

Obesity, which is mainly caused by positive energy imbalance and is associated with aging, has become a global health problem and increases the risk for type 2 diabetes mellitus, cardiovascular diseases, and many other diseases^[1]^. Studies have indicated that low-grade chronic inflammation characterized by immune cell infiltration and phenotypic changes in adipose tissue (AT) and other tissues occurs in obesity and may contribute to obesity-associated diseases^[2–9]^. Macrophages are the most abundant immune cells in AT, can change to classically activated (M1)- or metabolically activated-like phenotypes in obesity, and play an important role in AT inflammation by secreting proinflammatory cytokines, including tumor necrosis factor-alpha (TNF-α), interleukin-6 (IL-6), and interleukin-1β (IL-1β)^[10–17]^.

In addition to macrophages, lymphocytes, including T lymphocytes and B lymphocytes, are another type of immune cells in AT that play a crucial role in AT inflammation and may contribute to insulin resistance (IR)^[6,18–24]^. In this review, we summarize the current knowledge of T cells and B cells in AT and their potential roles in obesity-linked metabolic disease, aiming to provide a new perspective on targeting these immune cells to prevent obesity and related IR.

### T cells and B cells

T cells and B cells are both adaptive immune cells responsive to antigens. While T cells are responsible for cellular immunity mainly by producing cytokines or via cell interactions, B cells mediate humoral immunity mainly by producing antibodies.

Based on T cell receptors (TCR_s_), T cells can be categorized into αβT cells and γδT cells, both of which play a crucial role in immune functions^[25,26]^. The majority of T cells in most tissues are αβT cells, which express α and β TCR chains^[25]^. γδT cells possess a TCR consisting of γ and δ chains^[26]^. TCRαβ and TCRγδ share some similarities but are also different in several aspects. Although the variable (V) regions of TCRαβ and TCRγδ exhibit a similar structure, the distance between the immunoglobulin-like domains and the disulfide bond in the connecting peptide is longer in TCRγδ compared to TCRαβ. In addition to polar amino acids located in the transmembrane (TM) region, the sequence of other amino acids in the TM region of TCRγδ and TCR αβ differs greatly. TCRαβ can recognize foreign or mutated peptides presented on major histocompatibility complex (MHC) molecules, whereas the majority of TCRγδ does not recognize MHC molecules^[25]^.

Within the αβT cell population, CD4+ T cells can differentiate into T helper cells (Th) after antigen stimulation. Depending on stimuli and environment, CD4+ T cells can polarize into type 1 (Th1), type 2 (Th2), type 17 (Th17), or other types of T helper cells, which are different in numerous surface markers and released cytokines and therefore play different roles in inflammation [Table 1]. CD4+ T cells also contain a special regulatory subset known as regulatory T cells (Tregs), which are characterized by the expression of CD25 and Foxp3 and exhibit immunoregulatory functions mainly by inhibition of activation of conventional T cells, B cells, and natural killer (NK) cells. Tregs are involved in the maintenance of tissue homeostasis and self-tolerance, or contribute to the pathogenesis of some morbidities by downregulating immune responses^[21]^. Of the αβT cell population, CD8+ T cells predominantly mediate cell killing by secreting granzymes and perforin and are therefore also known as cytotoxic T lymphocytes (CTLs). In addition, CD8+ T cells can mount immune responses through the secretion of cytokines.

Similar to T cells, B cells are heterogeneous and consist of several distinct subsets. Broadly, B cells have been identified as B1, B2, and regulatory B cells (Bregs), which differ in originations, phenotypes, locations, and functions^[27,28]^. B1 cells primarily originate from the fetal liver and can be further classed into B1a and B1b cells, which are both CD19^high^, B220^−/low^, IgM^high^, IgD^low^, CD23^−^, CD43^+^, and CD1d^mid^, but different in CD5 with B1a being CD5^+^ and B1b being CD5^−[28]^. B1 cells are abundant in mucosal tissues, peritoneal cavities, omentum, and fat pads near the peritoneal cavity^[29,30]^. B2 cells are mainly derived from the bone marrow and are CD19^+^, B220^+^, CD21^high^, CD43^−^, and CD5^−[27,28]^. B2 cells constitute the major B cell population in secondary lymphoid organs and play a pivotal role in adaptive immune responses^[27,28]^. In contrast, Bregs primarily function to restrain immune responses by producing cytokines such as IL-10^[27,28]^.

### T cells in adipose tissue

In obesity and IR conditions, the proportions of Th1 cells are increased, whereas Th2 and Tregs are decreased in both humans and mice^[6,19,21–23,31]^. Animal studies indicated that Th1 cells and IFN-γ, the signature cytokine of Th1 cells, promote and Th2 cells and Tregs protect against IR in obesity [Table 1]^[6,19,21,23,32]^.

#### Th1 subset

The proportion of AT Th1 cells and the Th1 signature cytokine, IFN-γ, highly correlate with body mass index^[23,33]^ and are positively associated with AT inflammation and IR both in mice and humans^[19,34]^. The accumulation and polarization of Th1 cells in AT in obesity may be induced by the increased expression of class II major histocompatibility complex (MHC II) and costimulatory molecules on macrophages and adipocytes^[34–36]^. MHC II on either macrophages or adipocytes is sufficient to promote Th1 cell polarization and IFN-γ production^[35,36]^. In addition, AT macrophage- or dendritic cell-released IL-12 promotes Th1 differentiation and IFN-γ expression via activating signal transducer and activator of transcription 4 (STAT4)^[37]^.

Ablation of Th1 cells or IFN-γ in mice attenuates obesity-linked AT inflammation and IR, supporting a promoter role of Th1 cells and IFN-γ in AT inflammation and IR^[19,32,38]^. Mechanistically, Th1 cells may adversely regulate adipocyte or preadipocyte metabolism including impairing insulin signaling possibly via IFN-γ^[19,20,39]^. Th1 cells and IFN-γ may also contribute to AT inflammation and IR by inducing recruitment and M1-like phenotypic changes of macrophages in AT with obesity^[19,32,40]^. Deficiency of IFN-γ or its signaling molecule, STAT1, inhibits M1-like macrophage recruitment and TNF-α levels in AT and improves IR with obesity^[32,40]^. In addition, CD40L (CD154) expressed on Th1 cells may contribute to the accumulation of M1-like macrophages and the production of proinflammatory cytokines via interaction with CD40 expressed on macrophages in obese mice. CD40L deficiency in mice attenuates obesity-linked AT inflammation and hepatic steatosis and increases systemic insulin sensitivity^[41]^.

#### Th2 subset

Th2 cells can be identified by the expression of cytokines such as IL-4, IL-5, IL-9, and IL-13 and induce macrophage polarization into M2 phenotypes^[42]^. The proportion of Th2 cells is decreased in AT of obese humans and mice^[43]^. A previous study revealed that after adoptive transfer into obese T cell-deficient mice, CD4+ T cells from wild-type mice polarized into Th2 cells, which were associated with reversal of enhanced weight gain and IR in recipient T cell-deficient mice. In contrast, the transfer of T cells from Stat6-deficient mice, which have impairment in Th2 cell polarization, did not have these effects^[23]^. These data support a protective role of Th2 in the development of obesity and its related IR. Th2 cells and related cytokines may protect against obesity and metabolic complications by directly regulating adipocyte metabolism or by impacting other immune cells, such as M2 macrophages and eosinophils, both of which may have beneficial effects on obesity-related metabolism^[40,44,45]^.

#### Th17 subset

Th17 cells can be distinguished from other T cell subtypes by expression of IL-17^[46]^. IL-17 interacts with IL-17 receptor (IL-17R) expressed on other immune cells and epithelial cells and activates several signaling cascades such as NFκB, mitogen-activated protein kinases (MAPKs), and the CCAAT-enhancer-binding proteins (C/EBPs) cascades in these cells to produce inflammatory molecules. Th17 cells have been implicated in various autoimmune disorders and inflammation^[47]^. However, the role of Th17 cells in obesity and IR remains largely unexplored. An elevated proportion of Th17 cells is observed in AT, peripheral blood, spleen, and lymph nodes in both humans and mice with obesity^[48,49]^. The accumulation of Th17 cells in AT positively correlates with AT inflammation and IR, and studies in mice with the absence of IL-17 support the proinflammatory role of IL-17 in AT^[50]^. IL-17 may promote AT inflammation but inhibit adipocyte differentiation through TANK-binding kinases 1 (TBK1) and I-kappa-B kinase epsilon (IKBKE)^[50]^.

In AT, Th17 cells interact with and are regulated by other immune cells and adipocytes^[51,52]^. A distinct subset of dendritic cells characterized by being CD11c^high^F4/80^low^CX3CR1^+^ has been shown to correlate with Th17 differentiation in AT^[53]^. Adipose-derived stem cells (ASCs) from human subjects with obesity have been demonstrated to enhance IL-17 release by Th17 cells but inhibit the expression of IFN-γ and TNF-α by Th1 cells^[54]^. Furthermore, dysregulation of Tregs in obesity may contribute to the increase in Th17 in AT. Under healthy conditions, Th17 and Tregs cells are balanced; however, imbalance occurs in inflammatory conditions such as obesity and IR^[55]^. Th17 cells are susceptible to suppression by naïve and memory Tregs, which inhibit the production of IL-17, IL-22, and CXCL8^[56]^. Rab4b, a small GTPase governing endocytic trafficking in T cells, exhibits decreased expression in individuals with obesity, which may also contribute to the elevation of Th17 cells and reduction of Tregs within AT in obesity^[57]^.

#### Tregs

Tregs are usually a small portion of CD4+ T cells but are enriched in visceral AT (VAT) in lean conditions^[21,58]^. VAT enrichment of Tregs shows sexual dimorphism, with more Treg enrichment in male than female VAT^[58]^. AT Tregs exhibit elevated expression levels of CTLA-4, GITR, OX40, peroxisome proliferator-activated receptor (PPAR)-γ, and IL-10^[59]^. Obesity diminishes accumulation of Tregs in both VAT and subcutaneous AT (SAT) in mice and humans^[21,59]^ and changes VAT Treg signature. Depletion of Tregs in mice leads to increased gene expression of inflammatory mediators, including TNF-α, IL-6, and CCL5, and impaired metabolic signaling pathways within VAT, and expansion of Tregs improves insulin sensitivity in mice fed high-fat diet (HFD)^[21,60]^, supporting a protective role of Tregs in AT inflammation and IR associated with diet-induced obesity.

Tregs also participate in the regulation of adipocyte browning^[61]^. Brown AT or white AT browning facilitates nonshivering thermogenesis, representing a capacity for energy expenditure and holding potential for the treatment of obesity^[62]^. A unique subset of Tregs characterized by the expression of CD73^hi^ST2^lo^ in AT exerts IR-improving effects by promoting white AT beiging through the augmentation of adenosine production^[63]^.

Although the mechanisms responsible for the enrichment and function of Tregs in lean VAT have not been fully elucidated, several factors are considered crucial for Tregs accumulation in AT. PPAR-γ, the “master regulator” of adipocyte differentiation, is an essential regulator of the phenotype and function of Treg accumulation in VAT and contributes to Treg upregulation in conjunction with Foxp3. In obesity, the phosphorylation of PPAR-γ at Ser273 leads to the disappearance of this VAT Treg signature^[59,64,65]^. The enzyme hydroxyprostaglandin dehydrogenase (HPGD), which exhibits high expression levels in VAT Tregs, plays a pivotal role in maintaining VAT homeostasis and metabolic regulation and contributes significantly to the suppressive capabilities of VAT Tregs, which are partially induced by PPAR-γ^[66]^. Furthermore, adipocytes and other immune cells within AT also contribute to the accumulation, phenotype, and function of VAT Tregs. MHCII molecules are highly expressed on adipocytes and negatively correlated with Tregs in AT. The specific knockout of MHCII in adipocytes promotes Treg accumulation and M2-like macrophage polarization, possibly by inhibiting IFN-γ production in Th1 cells^[67]^. Costimulatory B7 molecules (CD80 and CD86) on antigen-presenting cells (APCs) may be important in maintaining Tregs in AT. CD80/CD86 double knockout in mice reduces AT Tregs, with enhanced AT inflammation and IR, while adoptive transfer of Tregs effectively mitigates IR and AT inflammation in CD80/CD86 double-knockout mice^[68]^. In addition, ST2, the IL-33 receptor, is highly expressed on VAT Tregs from humans and mice; IL-33 drives VAT Treg proliferation and is able to rescue VAT Treg numbers in obese mice, along with improving AT inflammation and IR^[69,70]^.

In contrast to the changes and role in diet-induced obesity, AT Tregs are increased with aging and may play an adverse role in age-associated immune responses and IR.^[71]^

#### CD8+ T cells

CD8+ T cells increase early and mainly accumulate in VAT in obesity^[22]^ and may participate in the progression of obesity-associated AT inflammation and IR^[18,19,22]^. Along with macrophages, CD8+ T cells participate in crown-like structure formation^[22,36]^. In obesity, AT CD8+ T cells polarize into an effector memory phenotype, with elevated expression of IFN-γ and granzyme B^[18,22]^. The accumulation and activation of CD8+ T cells may be induced by elevated IL-12 and IL-18 in obese AT^[18]^. Similar to Th1 cells, CD8+ T cells promote AT inflammation and IR^[18,22]^. CD8+ T cell deficiency in mice improves IR in obesity, associated with reduced macrophage infiltration and decreased M1-like macrophage recruitment^[22,72]^. In addition, blocking CD4+ and CD8+ T cell activation in mice with anti-CD40L antibody reduces weight gain, mitigates VAT inflammation, and alleviates obesity-induced IR, also supporting the role of T cell activation in the development of obesity and IR^[73,74]^. CD8+ T cells may contribute to the development of obesity and IR through inhibition of beige adipogenesis^[72]^.

#### γδ*T cells*

Similar to αβT cells, γδT cells accumulate within AT during obesity and play a role in AT inflammation and macrophage recruitment^[75]^. Mice with a deficiency of γδT cells have reduced M1-like macrophage accumulation and increased M2-like macrophage enrichment in VAT^[75]^. Upon activation, γδT cells mainly function through the production of cytokines and growth factors^[76]^. γδT cells are one major source of IL-17A in AT^[75]^, thereby contributing to AT inflammation, adipogenesis, and glucose metabolism. γδT cell-secreted IL-17 may also promote AT sympathetic innervation and thermogenesis through the IL-17 receptor C/TGFβ1 pathway in adipocytes^[77]^. Further, based on the BTB-POZ transcription factor, PLZF, γδT cells can be distinguished into two distinct populations with differences in IL-17A production^[78]^. Mice lacking γδT cells or IL-17A exhibit a low abundance of ST2+ Tregs and IL-33 in VAT and have impaired capacity to regulate core body temperature when exposed to cold^[69,78]^, supporting a role of AT resident γδT cells in the maintenance of AT immune homeostasis and control of body temperature.

#### NKT cells

Natural killer T (NKT) cells are characterized by the co-expression of NK cell markers (NK1.1 or CD56) and T cell marker (αβTCR)^[79]^. NKT cells primarily identify glycolipid antigens presented by the MHC class I-like molecule CD1d and can be categorized into two main types: type I and type II NKT cells^[6]^. Both NKT subtypes can produce Th1 and Th2 cytokines such as IFN-γ and IL-4 and contribute to the regulation of adaptive immunity. Type I NKT cells express the invariant TCRα (Vα14-Jα18 in mice, Vα24-Jα18 in humans) and are also named invariant NKT (iNKT)^[6]^. While some initial studies indicated that obesity in mice increased VAT NKT cells, including iNKT, and that NKT cells may promote obesity-linked AT inflammation^[80–82]^, others reported that iNKT cells are highly enriched in AT of lean humans and mice and are decreased in AT of obese individuals^[79,83,84]^.

The presence and activation of iNKT cells in AT depend on their interaction with CD1d molecules expressed on adipocytes^[79]^. In normal conditions, adipocytes with high CD1d expression act as APCs that present lipid antigens to iNKT cells, thereby sustaining iNKT cell populations and promoting their activation within AT^[84]^. Obesity is associated with a decrease in CD1d expression in both human and mouse AT, resulting in a reduction of iNKT cells in AT^[85]^.

AT iNKT cells have a unique transcriptional program and produce IL-2 and IL-10, which may promote M2-like macrophage polarization and control the proliferation and suppressive function of Tregs in AT^[86]^. While some studies showed that iNKT cell deficiency in mice did not impact weight gain^[81,86]^, with no effects on glucose tolerance^[86]^ or with improved insulin resistance^[81]^, another study showed that mice with iNKT cell deficiency had increased weight gain and exacerbated insulin resistance, along with proinflammatory macrophage infiltration^[83]^. The reasons for the data discrepancy are not clear. Differences in housing conditions and environment may have contributed to the discrepancy.

### B cells in adipose tissue

Similar to T lymphocytes, B cells infiltrate VAT and undergo functional and phenotypic changes in response to diet-induced obesity and IR^[24,87–90]^. B1 cells negatively correlate with AT inflammation and IR, whereas B2 cells are positively associated with AT inflammation and IR [Table 1]^[24,89,90]^.

#### B1 cells

Of the B1 cells, B1a cells are recognized as the primary producers of natural IgM antibodies, while B1b cells are responsible for initiating adaptive humoral immune responses against T cell-independent antigens^[91]^. In AT, B1 cells constitute a small portion of B cells, accounting for ~20%–30% of total B cells^[24,29,90]^. Reports on changes in AT B1 cells in obesity were not consistent, with some studies^[29,90]^ showing reductions but another study^[24]^ showing slight increases in AT B1 cells in mice with obesity. B1a cells are identified as the major producers of B cell-derived IL-10, which exerts anti-inflammatory functions in obesity-induced AT inflammation^[29]^. Adaptive transfer of B1a cells or IL-10 rapidly improves insulin resistance and glucose tolerance, supporting the protective role of B1a and IL-10 in IR^[29]^. B1b cells in AT reduce cytokine production by M1-like macrophages, and adoptive transfer of B1b cells exerts anti-inflammatory effects in AT^[89]^. Further, B-1b cells protect against the development of obesity-associated glucose intolerance in an IgM-dependent manner^[89]^. In addition, B1 cell-produced IgM antibodies exhibit cross-reactivity with membrane lipids and circulating oxidized low-density lipoprotein (oxLDL)^[92]^. The neutralization of oxLDL by natural IgM antibodies has been demonstrated to protect against inflammation associated with atherosclerosis^[93]^.

#### B2 cells

B2 cells produce specific antibodies in response to T cell-dependent antigens^[94]^. Depending on the microenvironment, B2 cells also possess the capacity to differentiate into effector cells, which can produce proinflammatory cytokines such as IFN-γ and IL-12 and anti-inflammatory cytokines including IL-10 and IL-4^[95]^. In AT, B2 cells account for ~70%–80% of total B cells and are significantly increased in VAT of mice with HFD-indued obesity^[24,90]^. B cell deficiency in mice reduces obesity-induced AT inflammation and improves IR, with impacts on weight gain, and adoptive transfer of AT B2 cells from wild-type mice restores AT inflammation and insulin resistance in mice with B cell deficiency^[24,90]^, indicating a promoting role of B2 cells in the development of AT inflammation and IR in obesity. B2 cells may promote IR and AT inflammation by activating macrophages and T cells through cytokine production and antigen presentation and by producing pathogenic IgG antibodies^[24,96]^. The recruitment and activation of B2 cells in AT in obesity may be mediated by the interaction of leukotriene B4 (LTB4) and its receptor LTB4R1, which is highly expressed on AT B2 cells^[90]^.

#### Breg cells

Bregs are characterized by producing IL-10 and transforming growth factor-β (TGF-β) and have anti-inflammatory effects^[27,28,97]^. However, various other B cell subsets, including B1a and B1b, are able to produce IL-10^[27–29]^. Nishimura *et al.* reported that B cells in AT, but not in the spleen, in old normal chow-fed mice express IL-10 and that these IL-10-expressing B cells in AT are distinct from other known IL-10-expressing B cell subsets and are considered Bregs^[97]^. Diet-induced obesity in mice reduces IL-10 expression in AT B cells^[97]^. The frequencies of Bregs are also diminished in AT of individuals with overweight and obesity compared to individuals with normal weight^[98]^. B cell-specific IL-10 deletion aggravates AT inflammation and IR in obese mice, whereas adaptive transfer of AT Bregs ameliorates these effects^[97]^, supporting a protective role of IL-10-expressing Bregs in obesity-linked inflammation and IR.

#### T-bet+ B cells

T-bet+ B cells are a subset of B cells that express T-bet and CD11c but lack CD21 and CD23, and expand during chronic inflammation^[99]^. The frequencies of T-bet+ B cells are elevated in AT of humans and mice with obesity^[100,101]^. The increased frequencies of AT T-bet+ B in obesity rely on iNKT cells and TLR7 stimulation^[100–102]^. Mice with ablation of T-bet in B cells are protected from AT inflammation and IR with obesity, while the adaptive transfer of T-bet+ B cells aggravates IR in obesity, suggesting a proinflammatory and pathological role of T-bet+ B cells in obesity-linked inflammation and metabolic complications^[100]^. T-bet+ B cells may contribute to inflammation through the production of IgG2c during obesity. Along with the reductions in inflammatory cytokines and macrophages in AT, mice with ablation of T-bet in B cells have reduced serum levels of IgG2c^[100]^.

### Conclusion and perspective

Obesity is mainly caused by an energy imbalance between energy intake and energy expenditure and is associated with aging^[103]^. It has been well recognized that obesity is associated with low-grade chronic AT inflammation, with changes in the numbers and phenotypes of various types of immune cells^[4–9]^. While macrophages are the immune cells first reported in AT^[10,17]^, lymphocytes including T cells and B cells also reside in AT and undergo numeric and phenotypic changes in obesity^[20,24]^. Obesity increases CD8^+^ effector memory T cells, CD4^+^ Th1 cells, and B2 cells, but reduces Treg and Th2 cells, in AT^[18,19,21–24,90]^.

Many studies mainly performed in rodent models have demonstrated that AT inflammation and immune cells may play a role in the development of obesity-associated metabolic complications, including IR and type 2 diabetes, through various mechanisms. Therefore, efforts targeting immune cells and inflammation have been explored to prevent and treat obesity-related diseases^[104–106]^. The classical generic anti-inflammatory drugs, salicylates, have been shown to lower blood glucose levels in humans with obesity and/or type 2 diabetes^[104,107–109]^. Another generic anti-inflammatory drug, methotrexate, reduces hemoglobin A1c levels in patients with rheumatoid arthritis^[110]^. Several large clinical trials have shown the efficacy of therapies targeting inflammation in the prevention of atherosclerotic cardiovascular diseases over the past few years^[111–113]^. However, targeting inflammation or immune cells has not proven very successful for the prevention and treatment of obesity-related metabolic disease in large clinical trials. A significant barrier to the development of effective immune therapies for obesity and its metabolic complications is our limited knowledge of the mechanisms that regulate immune responses specific to obesity and the precise pathways through which immune cells influence metabolism.

The JAK/STAT pathways play critical roles in inflammation and have recently been active therapeutic targets for inflammatory diseases. Several JAK inhibitors have been approved by the US Food and Drug Administration (FDA) for the treatment of inflammatory diseases such as rheumatoid arthritis and psoriasis^[114]^. The JAK/STAT pathways are also activated early and persistently in AT with obesity and may contribute to AT inflammation and IR in obesity^[39,40,115]^. Therefore, we and others tested the effects of targeting the JAK/STAT pathways on immune and metabolic phenotypes in mouse models of HFD-induced obesity. Of note, treatment with baricitinib, an FDA-approved JAK1/JAK2 inhibitor for rheumatoid arthritis, reduces Th1 cells in AT and improves insulin sensitivity in mice fed HFD^[34,116,117]^. A phase 2 randomized controlled clinical trial involving 129 participants showed that baricitinib treatment (for 24 weeks) of humans with type 2 diabetes and diabetic kidney disease reduced inflammation, improved renal functions, and lowered hemoglobin A1c levels^[118]^, indicating a potential of repurposing FDA-approved medications to treat obesity- and/or diabetes-related complications. Another example is auronofin, another FDA-approved rheumatoid arthritis drug, which exerts beneficial effects on obesity-associated metabolic abnormalities in mouse models of diet-induced obesity^[119]^. Future studies will need to focus on deeper insights into the roles and mechanisms of immune cells in metabolic diseases, which could potentially unveil innovative paths for identifying new pharmacological targets and agents for the prevention and treatment of metabolic diseases, including type 2 diabetes.

## Figures and Tables

**Table 1.** Major AT lymphocytes and their roles in obesity

Cell phenotypes				Markers		Major cytokines secreted	Changes in obesity and role in IR

αβ T	CD4+	T helper^[120–123]^	Th1	Surface	CD3^+^, CD4^+^, CD8^−^, CCR1^+^, CCR5^+^, IL-12 R β2^+^, IL-27 R α^+^, IFN-γ R2^+^, IL-18 Rα^+^, and CXCR3^+^	INF-γ, IL-2, TNF-α, and TNF-β	Increase in obesity; promote IR^[19,32,34–36,38,40]^
				Intracellular	STAT1^+^, STAT4^+^, and T-bet^+^		
			Th2	Surface	CD3^+^, CD4^+^, CD8^−^, CCR3^+^, CCR4^+^, CCR8^−^, CD14^−^, CD19^−^, CXCR4^+^, IL-4 R α^+^, IL-17RB^+^, ST2/IL-33R^+^, and TSLP R^+^	IL-4, IL-5, IL-9, IL-10, IL-13, and IL-21	Decrease in obesity; alleviate IR^[23,40,44,45]^
				Intracellular	GATA-3^+^, IRF4^+^, STAT5^+^, and STAT6^+^		
			Th17	Surface	CD3^+^, CD4^+^, CD8^−^, CCR4^+^, CCR6^+^, CD14^−^, CD19^−^, IL-1 RI^+^, IL-6 R α^+^, IL-21 R^+^, IL-23 R^+^, and TGF-β RII^+^	CCL20, IL-17A, IL-17F, IL-21, and IL-22	Increase in obesity; promote IR^[48–50]^
				Intracellular	Batf^+^, IRF4^+^, RORα^+^, RORC2^+^, and STAT3^+^		
		Treg^[124,125]^		Surface	CD3^+^, CD4^+^, CD5^+^, CD14^−^, CD19^−^, CD25^+^, CD39^+^, CD103^+^, CD127^low^, CTLA-4^+^, folate receptor 4^+^, GITR^+^, CD223^+^, LAP^+^, LRRC32^+^, BDCA-4^+^, OX40^+^, and CD62^+^	Galectin-1, IL-10, IL-35, and TGF-β	Decrease in diet-induced obesity, but increase with aging; alleviate IR in diet-induced obesity^[59,64,65,67]^, promote obesity and IR with aging^[71]^
				Intracellular	FoxP3^+^ and STAT5^+^		
	CD8+^[126]^			Surface	CD3^+^, CD4^−^, CD8^+^, CD28^+^, CCR4^+^, CCR6^+^, CD69^+^, CD103^+^, and KLRB1^+^	TNFα, INF-γ, IL-2, IL-4, IL-5, IL-9, IL-10, and IL-17	Increase in obesity; promote IR^[18,22]^
				Intracellular	TBX21^+^, GATA3^+^, IRF4^+^, and RORC^+^		
	NKT^[127]^			Surface	CD3^+^, CD4^+/−^, CD8^+/−^, CD56^+^, CD161^+^, CD1d^+^, NK1.1^+^ (in mice) and CD94^+^	IFN-γ and IL-4	Increase in obesity; promote IR^[80–82]^ Decrease in obesity; alleviate IR^[79,83,84]^
				Intracellular	T-bet and Eomes		
γδ T^[26]^				Surface	Vγ7^+^, Vγ1^+^, Vγ4^+^, Vγ5^+^, Vγ6^+^, CD27^+/−^, CD45RB^+^ and NK1.1^+^	IL-17A	Increase in obesity; promote IR^[75]^ Increase in obesity; alleviate IR^[77,128]^
B Cells	B1^[28,129]^			Surface	CD19^high^, B220^−/low^, IgM^high^, IgD^low^, CD23^−^, CD43^+^, and CD1d^mid^	IL-10 and IgM	Decrease in obesity; alleviate IR^[24,29,90]^
	B2^[130]^			Surface	CD19^+^, B220^+^, CD21^high^, CD11b^low^, CD43^−^, and CD5^−^	IFN-γ, IL12, IL10, IL4 and IgG	Increase in obesity; promote IR^[24,90]^
	Breg^[130]^			Surface	CD1d^high^, CD5^+^, CD19^+^, CD40^+^, CD21^+^ CD24^+^, IgD^+^, and IgM^+^	IL-10, IL-35, and TGF-β	Decrease in obesity; alleviate IR^[27,28,97]^
				Intracellular	EBF-1^+^, E2A^+^, Oct2^+^, and Pax5^+^		
	T-bet+			Surface	CD19^+^, IgM^+^CD11c^+^ CD21^−^ and CD23^−^	IgG2a/c	Increase in obesity; promote IR^[100]^
				Intracellular	T-bet^+^		

## Data Availability

No original data were generated for this review article.
